# Topological changes of brain network during mindfulness meditation: an exploratory source level magnetoencephalographic study

**DOI:** 10.3934/Neuroscience.2022013

**Published:** 2022-05-07

**Authors:** Anna Lardone, Marianna Liparoti, Pierpaolo Sorrentino, Roberta Minino, Arianna Polverino, Emahnuel Troisi Lopez, Simona Bonavita, Fabio Lucidi, Giuseppe Sorrentino, Laura Mandolesi

**Affiliations:** 1 Department of Social and Developmental Psychology, University of Roma “Sapienza”, 00185 Rome, Italy; 2 Institut de Neurosciences des Systèmes, Aix-Marseille University, 13005 Marseille, France; 3 Institute of Applied Sciences and Intelligent Systems, CNR, 80078 Pozzuoli, Italy; 4 Department of Motor Sciences and Wellness, University of Naples “Parthenope”, 80133 Naples, Italy; 5 Institute for Diagnosis and Cure Hermitage Capodimonte, 80131 Naples, Italy; 6 Department of Advanced Medical and Surgical Sciences, University of Campania “Luigi Vanvitelli”, 80138 Naples Italy; 7 Department of Humanities, University Federico II, 80133, Naples

**Keywords:** functional connectivity, neuroplasticity, synchronization, graph theory, Vipassana Meditation

## Abstract

We have previously evidenced that Mindfulness Meditation (MM) in experienced meditators (EMs) is associated with long-lasting topological changes in resting state condition. However, what occurs during the meditative phase is still debated.

Utilizing magnetoencephalography (MEG), the present study is aimed at comparing the topological features of the brain network in a group of EMs (n = 26) during the meditative phase with those of individuals who had no previous experience of any type of meditation (NM group, n = 29). A wide range of topological changes in the EM group as compared to the NM group has been shown. Specifically, in EMs, we have observed increased betweenness centrality in delta, alpha, and beta bands in both cortical (left medial orbital cortex, left postcentral area, and right visual primary cortex) and subcortical (left caudate nucleus and thalamus) areas. Furthermore, the degree of beta band in parietal and occipital areas of EMs was increased too.

Our exploratory study suggests that the MM can change the functional brain network and provides an explanatory hypothesis on the brain circuits characterizing the meditative process.

## 1. Introduction

The meditative technique of Mindfulness Meditation (MM) allows one to develop an attitude of awareness of the present moment, whereby individuals perform a deliberate attempt to keep an open, curious and non-judgmental attitude [1],[2]. It is widely demonstrated that MM is associated with psychological wellbeing and mental health [3]–[6], probably correlated to a significant decrease in cortisol levels [7]. Recently, scientific evidence has also documented structural and functional effects of MM on the brain [8]–[10], suggesting that MM induces neuroplasticity. In this context, it has also been observed that MM provides beneficial, long-lasting effects in terms of brain connectivity [11],[12]. However, the role of psychological factors on brain connectivity organization should be clarified. Moreover, it has been shown that the brain connectivity of meditators changes as they meditate [13] as well as in the resting state [14],[15].

To understand the mechanisms underlying the effects of meditation, the study of brain functional connectivity is a suitable approach [16]. Indeed, it allows to study the widespread rearrangements in the communication among brain areas within the framework of graph theory [15],[17]–[21]. Representing brain areas and their pairwise interactions as nodes and links of a network, we obtain a handy tool for studying the rearrangements of the brain's functional organization [22].

Besides functional Magnetic Resonance Imaging (fMRI), relevant information on the functioning of the brain networks can be retrieved using neurophysiological techniques, such as electroencephalography (EEG) and magnetoencephalography (MEG), since they capture the electrical and magnetic signals, respectively, which arise from neuronal postsynaptic potentials. MEG, detecting the patterns of neural oscillations in specific frequency bands with highly accurate temporal and spatial resolution, allows one to investigate the interactions between brain regions [23].

MEG studies have demonstrated that during meditation large-scale networks undergo functional changes, such as the modulation of the dynamic balance between frontoparietal and Default Mode Network (DMN) in the alpha frequency band [24]. Moreover, a more efficient network communication was observed in the brain network of experienced meditators with respect to inexperienced ones [11], suggesting that the degree of expertise plays a key role in determining consolidated effects even during meditation practice [12]. Wong et al. (2015) showed greater global brain connectivity in the theta band in meditators than in controls, hypothesizing that the brain of meditators develops different topological features that are stable over time compared to people who do not practice meditation [25]. In this context, we have previously observed that experienced mindfulness meditators showed long-lasting topological changes in the right hippocampus during resting state condition, suggesting that MM might also have functional effects on prospective and spatial memory [26]. Furthermore, a study comparing meditation phase and resting state in expert meditators showed increased nodal degree of thalamus, posterior cingulate cortex, precuneus, medial prefrontal cortex, inferior parietal lobule, and dorsolateral prefrontal cortex, during the meditative phase [27].

Although many studies showed that the degree of experience in meditation practice is related to specific topological characteristics, the results are not univocal. This might depend on the different methodologies (fMRI, EEG, MEG), the multiplicity of paradigms, and the type of meditation. In particular, there are very few studies focusing on Vipassana meditation using MEG methodology. To overcome these limitations and to confirm the few previous results obtained with MEG, here we investigated the brain network topology of a group of experienced meditators (EM group) during meditative activity and compared their topological features with the ones of a group of individuals with no experience of meditation (NM group). Intuitively, the condition of MM for experienced meditators referred to a mental status and the use of a technique well known to them. On the contrary, for non-meditators the MM had no specific meaning, an experimental paradigm that we defined as “pseudo-meditation”. Hence, for each participant, we built a functional network where the links represent how two signals are synchronized based on source-reconstructed magnetoencephalography signals. To estimate synchronization we used the Phase Lag Index (PLI) [28]. Furthermore, to avoid potential biases induced by different edge densities or average degrees [29], we used the minimum spanning tree (MST) to build a functional network, obtaining topologically comparable measures [30].

## 2. Materials and methods

### 2.1. Participants

We acquired the magnetoencephalography signals of twenty-six EMs (eight males and eighteen females; mean age 42.6 years, ± SE 2.4) and twenty-nine NMs (nine males and twenty females; mean age 43 years, ± SE 1.98). EMs were trained in Vipassana Meditation and had an average of 6.41 (SE = ±1.489) years of meditation experience. All participants had been meditating in the last year for 1 hour or more, for at least five days a week. Exclusion criteria were: 1) major internal illnesses, 2) neurological or psychiatric illnesses, 3) left dominance, 4) participation in Vipassana retreats during last year. Initially, the EM and NM group consisted of twenty-nine and thirty-one participants respectively. However, one EM and two NMs were excluded from the study as their recording were very noisy [31]. In addition, two EMs were excluded from further analysis because one was left-handed and one had a neurological disorder. All participants were native Italian speakers and provided written informed consent that clarified the purpose of the study. The study was carried out in accordance with the Declaration of Helsinki and was approved by the “Comitato Etico Campania Centro” (Prot.n.93C.E./Reg. n.14-17OSS).

### 2.2. MEG system

The MEG system was developed by the National Research Council, Pozzuoli, Naples, at the Institute of Applied Sciences and Intelligent Systems “E. Caianiello” [31]. The system is equipped with 163-magnetometers and placed in a magnetically shielded room (ATB, Ulm, Germany) [32]. Acquisition, pre-processing, and source reconstruction were carried out as previously described [20]. Before the acquisition, four position coils were placed on the subject's head and digitalized using Fastrak (Polhemus®). The coils were activated, and localized, at the beginning of each registration segment. The subject was seated on a comfortable armchair placed in the shielded room. Electrocardiographic and electrooculographic signals were co-recorded to aid artefact removal [31]. After an anti-aliasing filter, MEG data were acquired with a sampling frequency of 1024 Hz. The signal was then filtered using a fourth order Butterworth IIR band-pass filter in the 0.5–48 Hz band.

### 2.3. Experimental procedures

The experimental protocol provided the MEG recording during the meditation phase. First, the brain signals of all participants were acquired in a resting condition with their eyes closed, twice for 2.5 minutes for a total of 5 minutes. After a short break of 5 minutes, the participants underwent a 10-minute MEG recording, with eyes closed, during the condition of meditation. During the five minutes-break, the participants remained in the MEG room. They could open their eyes and stretch legs and arms while remaining seated. For the recordings of the meditations, the following instructions were provided: “Close your eyes and relax. Try to reach a state of inner silence”. No further instruction was given during the 10 minutes recording. In this way, all participants, both EMs and NMs, applied this instruction according to their own experience. In conclusion, for EMs, this condition was a real phase of meditation, while for NMs, this phase represented a “pseudo-meditation”. The present study was not conducted in the single or double-blind mode. The purpose of the study was made explicit both to the participants (meditators and non-meditators) and to those who collected and processed the data.

### 2.4. Preprocessing

Firstly, starting from raw signals, we applied the Principal Component Analysis (PCA) to reduce environmental noise [32],[33]. Subsequently, an experienced operator visually inspected the channel signals and through manual procedure removed both channels with noisy signals and parts of the recording where the signals of all channels were noisy [31]. Through this procedure, we obtained a clean recording of variable duration for each participant. Further signal supervision was performed through Independent Component Analysis (ICA) [34], which was applied to remove physiological, cardiac (generally one component), and blinking (if present) artifacts from the MEG signals. After the cleaning procedure, the entire cleaned MEG recording was split into 8-seconds long segments (epochs). The length of 8 seconds is a trade-off between the need to have enough cleaned epochs and to obtain a reliable estimate of the connectivity measure [35]. So, each recording consisted of a different number of epochs, depending on the parts of the recordings that were cleared. However, to make a proper comparison between the two groups, we considered the same number of epochs for each participant based on the recording of the participant with the lowest number of epochs. We tested whether there were any differences in the number of epochs excluded between the EM and NM groups, and there were no statistical differences. We used the same exclusion criteria for both resting state and meditations recordings to establish the number of epochs available for the analysis of brain signals. Therefore, twelve and twenty-four epochs, respectively for resting state and meditation recordings, that did not contain artifacts (either system related or physiological) or strong environmental noise were selected. Starting from these, we estimated the connectivity measures.

### 2.5. Source reconstruction

All the processing related to the beamforming procedure has been done using the Fieldtrip toolbox [36]. Based on an MRI template, the volume conduction model proposed by Nolte [37] was considered and the Linearly Constrained Minimum Variance (LCMV) beamformer [38] was implemented to reconstruct time series related to the centroids of 116 regions-of-interest (ROIs), derived from the Automated Anatomical Labeling (AAL) atlas [39]–[41]. We decided not to include cerebellar areas (hence considering 90 ROIs) given their low reliability [42],[43]. We projected the time series along the dipole direction for each source that explained the most variance by means of singular value decomposition (SVD). Source time series were resampled at 512 Hz.

### 2.6. Connectivity and network analysis

The PLI was used to estimate functional connectivity [28], using BrainWave software [CJS, version 09.152.1.23, available from http://home.kpn.nl/stam7883/brainwave.html]. The PLI is based on the distribution of the differences of the instantaneous phases (∆Φ(t)) (derived from the Hilbert transform of the times series) and, for two-time series, is computed as:

PLI=|〈sign[sin(ΔΦ(tk))]〉| (1)

where “< >” indicates the mean value, “sign” stands for the signum function, “|.|” denotes the absolute value and “tk” are the samples. The phase difference is defined in the [−π, π] range. This measure is insensitive to volume conduction (at the cost of discarding true zero–lag interactions). PLI values range between 0 and 1, where 1 indicates perfect synchronization and 0 indicates nonsynchronous activity. Hence, by computing the PLI for each couple of brain regions, we obtained a 90 × 90 weighted adjacency matrix for each epoch, in all of the frequency bands: delta (0.5–4.0 Hz), theta (4.0–8.0 Hz), alpha (8.0–13.0 Hz), beta (13.0–30.0 Hz) and gamma (30.0–48.0 Hz). A weighted adjacency matrix was interpreted as a network, where the 90 areas of the AAL atlas are represented as nodes, and the PLI values form the weighted edges. Thereafter, the MST was calculated for each adjacency matrix in each frequency band. Since we were interested in the strongest connections for the construction of the MST, the edge weight was defined as 1/PLI. In fact, Kruskal's algorithm [44] first ranks the links in ascending order and then constructs the network by adding one link at a time, discarding links that would form a loop. The algorithm proceeds until all nodes are connected resulting in a loop-less graph with N nodes and M = N − 1 links. To avoid some biases in traditional network analyses [29], we used the MST that allows for an unbiased topological interpretation of the results [30],[45].

Based on the MST, we calculated both global and nodal topological parameters [30],[46] in EM and NM groups during the meditation and pseudo-meditation phases. We calculated the leaf fraction, degree divergence, and tree hierarchy, as global parameters. The *leaf fraction* is defined as the fraction of nodes with a degree of 1 [46], indicating the integration of the network. A higher leaf fraction implies that the network tends toward a star-like topology, where the nodes are on average closer to each other with respect to a line–like topology. The *degree divergence* is a measure of the broadness of the degree distribution related to the resilience against attacks [30]. The *tree hierarchy* is defined as the number of leafs over the maximum betweenness centrality (see later). The idea behind this measure is that an optimal network should achieve efficient communication while avoiding hub overload. The tree hierarchy has been designed to quantify the balance between both features. For what concerns the nodal parameters, we calculated the *degree* and the *betweenness centrality* (BC) (both representing indirect measures of brain network integration) for each node, to determine if specific regions differed. In particular, the degree is the number of connections incident on a given node. The BC is defined as the number of shortest paths passing through a given node over the total of the shortest paths of the network [47]. These metrics were calculated for each epoch and subsequently averaged across epochs for each subject separately.

### 2.7. Statistical analysis

All statistical analyses were performed in Matlab (Mathworks®, version R2013a). To compare the two groups, for each frequency band and each parameter, we used permutation testing [48], where the null distribution for between-group differences is derived from the data. In detail, assuming no group differences, the labels of the subjects were permuted 10000 times. Each time, the differences between the averages of the two groups were computed, obtaining the null distribution for between-group differences. Such distribution was used to define the statistical significance of the observed difference between the two groups. We used the False Discovery Rate (FDR) [49] to correct for multiple comparisons. A significance level of p ≤ 0.05 after FDR correction was used. Finally, we tested whether the results were stable when randomizing the choice of epochs one hundred times (see supplementary information (SI) for more details).

## 3. Results

**Figure 1.**
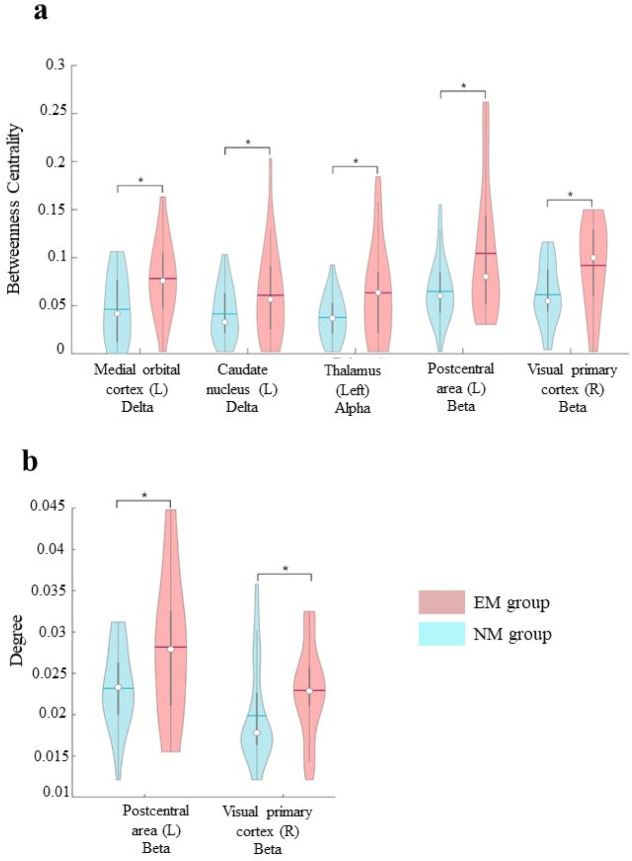
Comparison between Expert Meditators (EM) and individuals who had no previous experience of any type of meditation (NM) during the meditation phase. The violin plots refer to the topological changes in betweenness centrality (a) and degree (b) in delta, alpha and beta frequency bands occurring in specific cerebral areas. In both plots, the mean is represented by the horizontal line inside each plot. Significance p value: * = *p*_FDR_ < 0.05.

To analyse the possible effects of MM as compared to a condition of a nonspecific mental commitment, we compared the brain topology of EMs with that of NMs during the meditative experience (Figure 1a, b). Since no statistically significant differences were found between “pseudo-meditation” and resting-state in the NM group (data not shown), values obtained in the “pseudo-meditation” condition represented the baseline to interpret the trend of our results. We showed a statistically significant increase in the BC of the medial orbital cortex (pFDR = 0,027) and caudate nucleus (pFDR = 0,027) in the delta frequency band, both on the left hemisphere and in the left thalamus in the alpha frequency band (pFDR = 0,027). In the left postcentral area and in the right visual primary cortex both the BC (pFDR = 0,018 and 0,018 respectively) and the degree (pFDR = 0,045 and 0,045 respectively) were increased in the beta frequency band. No statistically significant results were obtained in other brain regions and frequency bands. Similarly, the global parameters were not significantly different in any frequency band. Furthermore, to assess the robustness of the results, the permutation test was repeated one hundred times, randomly choosing twenty-four different epochs per subject at each iteration (see SI, Figure S1, and S2). The analysis showed a p-values distribution tendency towards statistical significance.

## 4. Discussion

We have previously shown that the brain topology of experienced mindfulness meditators during resting state is different from age, sex, and education matched controls [26]. The present study aims to investigate the brain topology of the same cohort during the meditation phase, comparing it with the topology of individuals who had no previous experience of any type of meditation.

Our preliminary results show that the brain topology during the meditation phase of EMs is different from that of individuals not accustomed to meditation and are in accordance with previous studies [11],[24]. In particular, in EMs during the meditation phase a wide range of changes occur, involving the BC in delta, alpha and beta bands, encompassing frontal, parietal and occipital cortical areas, the caudate nucleus, and the thalamus (Figure 1a). The widespread change of the brain topology observed in the EMs during meditation suggests that to enter the meditative phase, they activate mechanisms that modify the role of specific areas.

The involvement of frontal areas such as the medial orbital cortex during meditation has been reported in multiple studies [8],[50]. It might reflect the goal of mindfulness meditators, which is to convey attention to their own body and to be focused on their own emotions, as well as to control possible interferences coming from the external environment. Indeed, the orbitofrontal cortex is generally associated with self-referential processing [51], and it is usually activated when subjects must choose if an adjective refers to themselves [52]. In line with this evidence, it is not surprising that the centrality of this area during meditation grows since the meditative state induces a shift of attention toward self and a greater awareness of one's internal states [53].

The higher BC in the left caudate nucleus in the delta frequency band might be linked to a mechanism whereby the subject improves the constant awareness of its own feelings. The caudate nucleus has been defined as “the interference control system”, responsible for maintaining attention and for low distractibility [53],[54], and this might be why it is involved in the more advanced phases of meditation when the condition of “awareness” must be maintained [6].

We also observed that the EMs have greater BC of the left thalamus in the alpha band. This could be related to the functional meaning of alpha rhythm. In fact, alpha oscillations regulated by the thalamus arise from increased internal attention [55],[56], which is expected in EMs. This oscillatory activity is in accordance with other evidence reporting increased alpha band activity in Vipassana meditation compared to naïve controls [50],[57],[58].

Moreover, it is important to focus on the centrality of the right visual primary cortex in the beta band, which is more central in EMs during meditation than pseudo-meditation of NMs (Figure 1b). It is known that the dorsal stream, the pathway that links the perception to the action [59], starts from this area. Moreover, the visual primary cortex is active during visual processing and during visual imagination tasks [60]. Hence, its activation is in accordance with other studies that observed visual experiences as flashes during meditation.

Overall, as suggested by the BC increase in multiple frequency bands, our results suggest that the brain network is more integrated in EMs than in NMs, which could facilitate information exchange between different brain areas. Our results partially agree with van Lutterveld et al. [11], who observed increased BC values in the alpha band only. This discrepancy could be due to both the individual characteristics of the sample and the experimental design. From a purely speculative perspective, increased integration of brain networks may underlie some of the beneficial effects of meditation, such as improved cognition.

However, it should be noted that we did not find in the EMs compared to the NMs any increase in the leaf fraction, a global topological parameter referring to the integration of the network. This inconsistency could be driven by the lack of a validated marker of the level of meditation reached by each subject. Therefore, further studies aimed at investigating the topological characteristics of brain networks as a function of the specific level of mediation reached are desirable.

Although several other studies on meditative techniques support our findings, our work must be considered in light of several limitations. Our results are preliminary and need to be replicated and confirmed. Furthermore, it must be kept into consideration that unforeseen elements, such as familiarity with the task (and not the task itself) or the ability to concentrate, may have played a significant role in determining the differences in brain connectivity. To conclude that the results are causally related to meditation, all other possibilities should be ruled out. Another limitation of the present study concerns the lack of psychological evaluation of the participants. In fact, it seems very interesting to understand if the topological changes found in meditators really depend on the meditation practice itself or the psychological well-being that meditation determines on the individual who practices it as evidenced by previous studies [61],[62]. Although with some limitations, the results of our study increase the knowledge about the topological changes correlated to Vipassana meditation helping to understand the effects of this practice on the brain.

## 5. Conclusions

In conclusion, our study highlighted that the meditative experience is associated with short-term topological changes modifying the centrality of specific areas, revealing a mental process regulated by specific task-dependent features that require active involvement. However, an integration of the results with a psychological assessment that considers the psychological factors correlated to meditation can help better understand the processes that regulate the topology of the brain networks and the relation between meditation and well-being.
