# Supplementary material for: Topological changes of brain network during mindfulness meditation: an exploratory source level magnetoencephalographic study
Source: AIMS Neurosci. 2022 May 7;9(2):250–63. doi: 10.3934/Neuroscience.2022013 (PMC9256519; doi:10.3934/Neuroscience.2022013)
Supplement: Supplementary file 1 [file neurosci-09-02-013-s001.pdf]

Neuroscience

AIMS Neuroscience, 9 (2): 250–263.
DOI: 10.3934/Neuroscience.2022013
Received: 24 September 2021
Revised: 21 March 2022
Accepted: 21 March 2022
Published: 07 May 2022

http://www.aimspress.com/journal/neuroscience

Research article

Topological changes of brain network during mindfulness meditation: an exploratory source level magnetoencephalographic study

Anna Lardone[1,†] and Marianna Liparoti[1,†], Pierpaolo Sorrentino[2,3], Roberta Minino[4], Arianna Polverino[5], Emahnuel Troisi Lopez[4], Simona Bonavita[6], Fabio Lucidi[1], Giuseppe Sorrentino[3,4,5,*] and Laura Mandolesi[7]

[1] Department of Social and Developmental Psychology, University of Roma Sapienza, 00185 Rome, Italy
[2] Institut de Neurosciences des Systèmes, Aix-Marseille University, 13005 Marseille, France
[3] Institute of Applied Sciences and Intelligent Systems, CNR, 80078 Pozzuoli, Italy
[4] Department of Motor Sciences and Wellness, University of Naples "Parthenope", 80133 Naples, Italy
[5] Institute for Diagnosis and Cure Hermitage Capodimonte, 80131 Naples, Italy
[6] Department of Advanced Medical and Surgical Sciences, University of Campania "Luigi Vanvitelli", 80138 Naples Italy
[7] Department of Humanities, University Federico II, 80133, Naples

[†] Anna Lardone and Marianna Liparoti contributed equally to the work

* **Correspondence:** Email: giuseppe.sorrentino@uniparthenope.it; Tel: +390815474805.

Supplementary Information

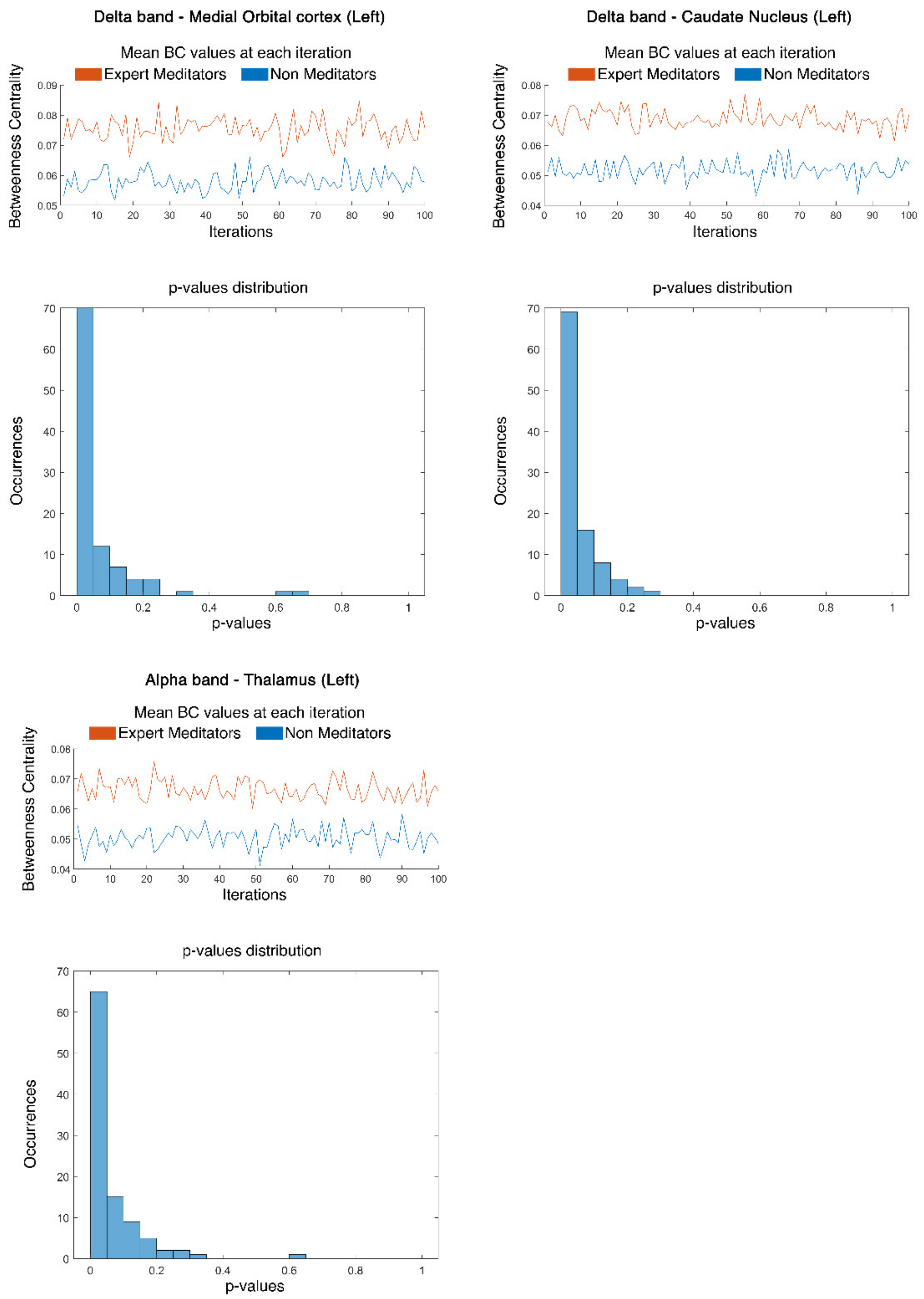

Figure S1. Randomization test on epochs in delta and alpha frequency bands.

The figure shows the randomization test on epochs for the significant results in delta and alpha band. The randomization consisted in repeating the permutation test between the two groups (i.e., Expert Meditators and Non Meditators) one hundred times, using 24 randomly chosen epochs per subject at each iteration. Specifically, the figure shows the randomization test for the beetweenness centrality of three brain regions: the Medial Orbital Cortex (Left) and the Caudate Nucleus (Left) in Delta band, and the Thalamus (Left) in Alpha band. For each randomization test, we reported two outcomes: on the top we showed the mean values of the two groups at each iteration, and on the

bottom we reported the p-values distribution of the permutation tests obtained by each iteration. Note that the first interval includes to the significant p-values (0 > p > 0.05).

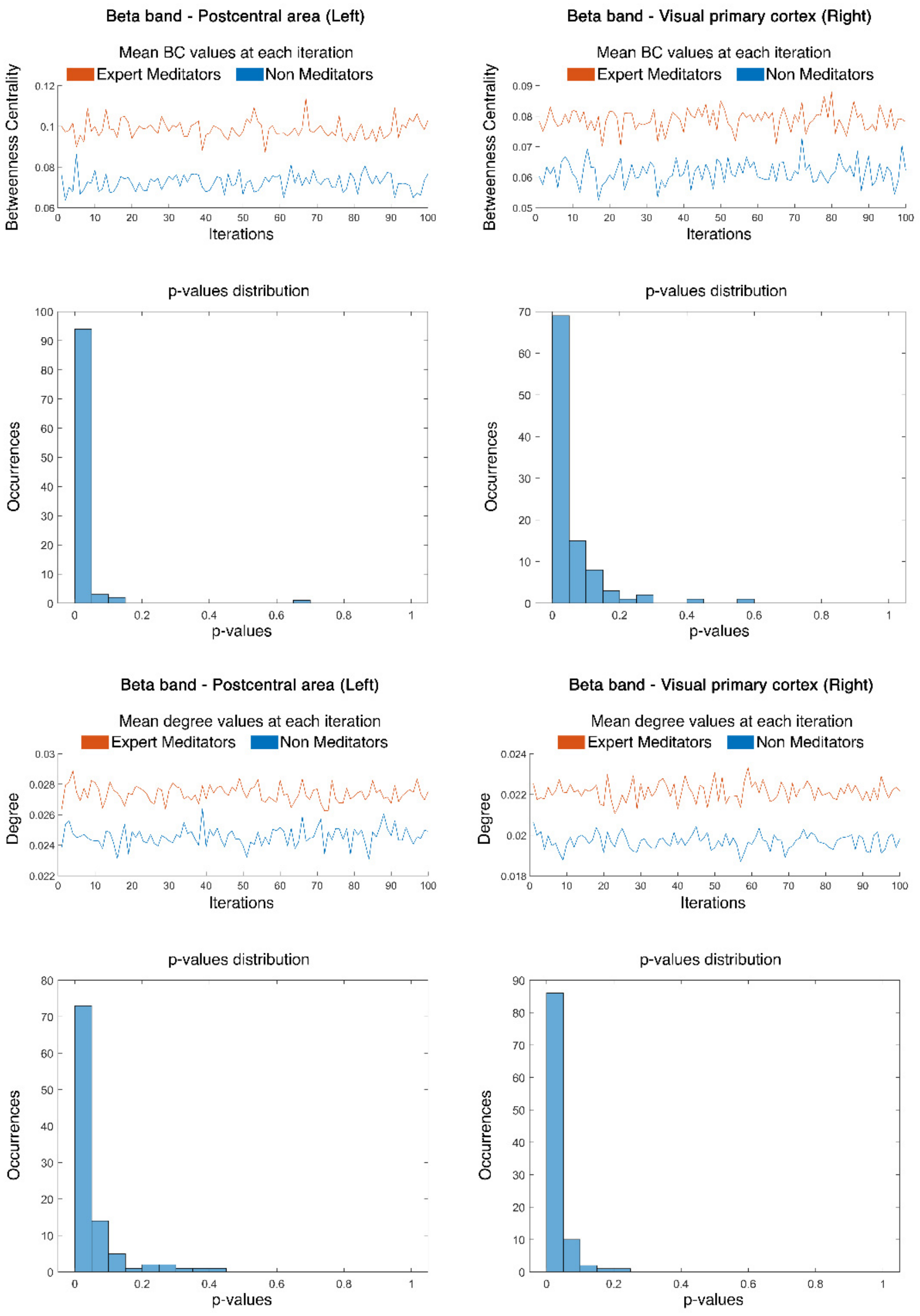

Figure S2. Randomization test on epochs in beta frequency band.

The figure shows the randomization test on epochs for the significant results in beta band. The randomization consisted in repeating the permutation test between the two groups (i.e., Expert Meditators and Non Meditators) one hundred times, using 24 randomly chosen epochs per subject at each iteration. Specifically, the figure shows the randomization test for the beetweenness centrality and the degree of two brain regions: the Postcentral Area (Left) and the Visual Primary Cortex (Left).

For each randomization test, we reported two outcomes: on the top we showed the mean values of the two groups at each iteration, and on the bottom we reported the p-values distribution of the permutation tests obtained by each iteration. Note that the first interval includes to the significant p-values ($0 > p > 0.05$).

AIMS Press

© 2022 the Author(s), licensee AIMS Press. This is an open access article distributed under the terms of the Creative Commons Attribution License (http://creativecommons.org/licenses/by/4.0)
